# A Novel Inducible Protein Production System and Neomycin Resistance as Selection Marker for *Methanosarcina mazei*

**DOI:** 10.1155/2012/973743

**Published:** 2012-07-19

**Authors:** Sebastian Mondorf, Uwe Deppenmeier, Cornelia Welte

**Affiliations:** Institute for Microbiology and Biotechnology, University of Bonn Meckenheimer Allee 168, 53115 Bonn, Germany

## Abstract

*Methanosarcina mazei* is one of the model organisms for the methanogenic order Methanosarcinales whose metabolism has been studied in detail. However, the genetic toolbox is still limited. This study was aimed at widening the scope of utilizable methods in this group of organisms. (i) Proteins specific to methanogens are oftentimes difficult to produce in *E. coli*. However, a protein production system is not available for methanogens. Here we present an inducible system to produce Strep-tagged proteins in *Ms. mazei*. The promoter p1687, which directs the transcription of methyl transferases that demethylate methylamines, was cloned into plasmid pWM321 and its activity was determined by monitoring **β**-glucuronidase production. The promoter was inactive during growth on methanol but was rapidly activated when trimethylamine was added to the medium. The gene encoding the **β**-glucuronidase from *E. coli* was fused to a Strep-tag and was cloned downstream of the p1687 promoter. The protein was overproduced in *Ms. mazei* and was purified in an active form by affinity chromatography. (ii) Puromycin is currently the only antibiotic used as a selectable marker in *Ms. mazei* and its relatives. We established neomycin resistance as a second selectable marker by designing a plasmid that confers neomycin resistance in *Ms. mazei*.

## 1. Introduction

Methanogenic archaea are strictly anaerobic microorganisms that play a key role in the global carbon cycle. In the anaerobic food chain they are responsible for the removal of acetate, methylated amines, and H_2_. The metabolism of methanogenic organisms has been studied extensively during the past decades. The process of methanogenesis is dependent on several unusual cofactors that are exclusively used in this pathway. Additionally, many proteins in methanogenic archaea possess unusual prosthetic groups that are limited to some members of the domain archaea (e.g., the tungsten cofactor of aldehyde: ferredoxin oxidoreductase), which are still not comprehensively described. One of the reasons for the limited knowledge is the inability to produce such enzymes in *Escherichia coli*. *E. coli* is very useful for the production of methanogenic enzymes that lack prosthetic groups. However, enzymes that possess properties not found in *E. coli*, or even in the bacterial domain, are difficult or impossible to produce in this well-characterized and extensively used bacterial protein production host. Consequently, a homologous protein production system in methanogens is needed for the production and purification of some methanogenic enzymes. The simplest way is to produce proteins fused to an affinity tag that allows purification via affinity chromatography. One potential overproduction host is *Methanosarcina (Ms.) mazei* that is a member of the family Methanosarcinaceae. Unlike most methanogens that are limited to growth on H_2_ + CO_2_ members of this family are well known for their metabolic versatility. Most members can grow on H_2_ + CO_2_, methylated amines, methanol, and acetate, and some can even grow on CO. This metabolic versatility is reflected in the relatively large genome size of *Ms. mazei* (4.2 Mpb, [1]) and its relatives. Such large genomes are an abundant source of genes encoding enzymes that are potential targets for homologous overproduction in *Ms. mazei.*

Genetical tools for *Ms. mazei* are constantly developed and improved. Members of the family Methanosarcinaceae are transformable by lipofection [2, 3] and suitable plasmids that are stable in *Ms. mazei* and its relatives exist [3]. Furthermore, it is possible to generate chromosomal deletion mutants using selection markers [4] or clean deletion systems [5, 6]. However, there are certain limitations: there is only puromycin available as a selectable marker and there is no protein production system allowing affinity purification of recombinant enzymes. Thus, this study aimed at widening the scope of genetical tools for *Ms. mazei*. A second antibiotic selectable marker is described, as well as a protein production system that for the first time allowed production and purification of a protein in *Ms. mazei*.

## 2. Materials and Methods

### 2.1. Bacterial Strains and Culture Conditions

For cloning of pASK-IBA3 and derivatives *Escherichia coli* DH5*α* was used. *uidA* was amplified from genomic DNA of *E. coli* K-12. For the modification of the methanogenic shuttle vector pWM321 [3] an *E. coli λ*pir strain was used that had the pir gene introduced into the genome by use of the *λ* phage [7]. Replication of pWM321 is dependent on the ori R6K and the copy number of the plasmid is dramatically increased if a strain harboring the gene encoding the pi protein is used. Both *E. coli* strains were cultivated in lysogeny broth (LB) containing 100 *μ*g mL^−1^ ampicillin for plasmid maintenance. *Methanosarcina mazei* Gö1 (DSM 7222) was grown in DSM medium 120 containing 150 mM methanol. Plasmids were introduced by lipofection [2] and cultures derived from single colonies were used for subsequent experiments. Depending on the resistance cassette, plasmid maintenance was ensured by the addition of 5 *μ*g mL^−1^ puromycin or 20 *μ*g mL^−1^ neomycin.

## 3. Generation of Plasmids

Restriction endonucleases, T4 DNA ligase, *Taq* DNA polymerase, and PCR reagents were purchased from Fermentas (St. Leon-Rot, Germany). Phusion DNA polymerase was purchased from New England Biolabs (Frankfurt am Main, Germany). Oligonucleotides were synthesized by Eurofins (Ebersberg, Germany). Routine molecular biological techniques were done according to Sambrook et al. [8].

The overproduction plasmid pSM01-uidA-Strep was constructed in a three-step process. First, the promoter p1687 was introduced into the methanogenic shuttle vector pWM321 [3]. Therefore, the promoter p1687 was amplified by PCR with chromosomal DNA from *Ms. mazei* using the primers 5′-TCTCGCGGCCGCTATGGGGTCCTAACCTCTTT-3′ and 5′-AATTCATATGATTCTCCTTTTGCCTTTTCAAC-3′ that introduced *Not*I and *Nde*I restriction sites (underlined). The PCR fragment was digested with these enzymes while pWM321 was digested with *Not*I and *Nhe*I resulting in compatible ends between the *Nhe*I and *Nde*I restriction digest. Both fragments were ligated. The resulting colonies were checked by colony PCR for the correct insertion of p1687 using the primers 5′-TCTCGCGGCCGCTATGGGGTCCTAACCTCTTT-3′ and 5′-TGTGGAATTGTGAGCGGATA-3′ and sequencing (StarSEQ, Mainz, Germany). In the next step, the reporter gene *uidA* fused to the coding sequence of the Strep tag should be cloned into pSM01. The *uidA* gene was amplified by PCR using the primers 5′- ATGGTAGGTCTCAAATGTTACGTCCTGTAGAAACCCCAA-3′ and 5′-ATGGTAGGTCTCAGCGCTTTGTTTGCCTCCCTGCTGCGG-3′ inserting *Bsa*I restriction sites (recognition sites underlined) and with genomic DNA of *E. coli* K-12 as template. The gene was cloned into pASK-IBA3 (IBA, Göttingen, Germany) using *Bsa*I that fuses the gene to the coding sequence of a Strep tag that will be connected to the C-terminus of the recombinant protein. Successful cloning was monitored by colony PCR using the primers pASK-for and pASK-rev (5′-CGCAGTAGCGGTAAACG-3′ and 5′-CGCCGCTACAGGGCGCGTGG-3′) and sequencing (StarSEQ, Mainz, Germany). The correctly cloned uidA-Strep fragment was excised with *Xba*I and *Nae*I and ligated into pSM01 that was cut with *Nhe*I and *Eco*RV. *Nhe*I and *Xba*I generate compatible ends whereas *Nae*I and *Eco*RV cut blunt ends. The correct cloning was monitored by colony PCR using the primers 5′-CCTGGCTTCCCACCCTGAC-3′ and pASK-rev. As a negative control, *uidA* was also cloned into pWM321 without the p1687 promoter using the same cloning strategy.

For the cloning of the neomycin resistance cassette, the *apH*-IIb gene was amplified by PCR from the plasmid pBBR-MCS1 [9] using the primers 5′-TATAACCAGGTTCAGAAGAACTCGTCAAG-3′ and 5′-TTAAAGGACCCGATGAGGATCGTTTCGCATG-3′. These primers introduced a *Ppu*MI site and a *Sex*AI site into the amplified fragment (underlined) that was cut with *Ppu*MI and *Sex*AI. *Sex*AI is inhibited by *dcm* methylation as occurring in the cloning strain so the Fast Digest variant (Fermentas, St. Leon-Rot, Germany) that was not inhibited by *dcm* methylation was used for the restriction of the plasmid pWM321. The plasmid was furthermore digested with *Rsr*II and together both restriction enzymes excise the puromycin resistance cassette (*pac*). Both fragments were ligated using T4 DNA ligase and the resulting colonies were screened by colony PCR using the primers 5′-TTAAAGGACCCGATGAGGATCGTTTCGCATG-3′ and 5′-CGCCGCATACAGTATTCTCA-3′. The correct insertion of the neomycin resistance cassette was checked by sequencing (StarSEQ, Mainz, Germany). The plasmid was named pWM321-neo.

## 4. Quantification of Promoter Strength

The relative promoter strengths of the promoter-reporter fusion constructs in *Ms. mazei *were investigated by measurement of *β*-glucuronidase activity (in Miller Units) essentially as described in [10]. *Ms. mazei* cultures (50 mL) harboring pSM01-uidA-Strep or pWM321-uidA-Strep were grown with 30 mM methanol up to an optical density at 600 nm of 0.15. Then protein production was induced by the addition of 50 mM trimethylamine. At different time points, 1 mL of the culture was harvested (8000 × g), resuspended in 100 *μ*L potassium phosphate buffer (40 mM, pH 7.0) that leads to the immediate lysis of the cells. The cell free lysate was used to determine *β*-glucuronidase activity by monitoring the production of *p*-nitrophenol from *p*-nitrophenyl *β*-glucuronide at 415 nm.

## 5. Protein Biochemical Methods

For protein purification, 1 L *Ms. mazei* harboring pSM01-uidA-Strep was grown to an optical density at 600 nm of 0.15 on 30 mM methanol. Then protein production was induced by the addition of 50 mM trimethylamine. After 30 h of induction the culture was harvested (8000 × g, 15 min) and resuspended in 5 mL buffer W (150 mM Tris, pH 8.0, 100 mM NaCl) that leads to the lysis of the cells. Affinity chromatography was performed as described by the manufacturer (IBA, Göttingen, Germany). Protein was quantified by the Bradford assay [11], and enzyme activity measurements were performed in analogy to the promoter-reporter fusions stated above. For the calculation of enzyme activity a molar extinction coefficient of 12 mM^−1 ^cm^−1^ for *p*-nitrophenol at 415 nm was used. Proteins were visualized after SDS-PAGE either by silver staining [12] or by Western blot and antibody detection. SDS-PAGE was done on a 12.5% (w/v) slab gel as described by Laemmli [13] with a 5% (w/v) polyacrylamide stacking gel. Samples were diluted in sample loading buffer (2% [w/v] SDS, 5% [v/v] *β*-mercaptoethanol, 50% [v/v] glycerol, 20% [v/v] collecting buffer pH 6.8, and 0.001% [w/v] bromophenol blue) and boiled for 5 min prior to application. Molecular mass was calculated by comparison to a molecular mass standard (Fermentas, St. Leon-Rot, Germany). For Western blot and antibody detection, proteins were blotted onto a nitrocellulose membrane using a semidry blotting device (Biozym, Hessisch Oldendorf, Germany). The membrane was blocked in PBS (140 mM NaCl, 2.7 mM KCl, 4.3 mM Na_2_HPO_4_, and 1.5 mM KH_2_PO_4_, pH 7.3) containing 5% milk powder for 1 h at room temperature. Then, the membrane was washed (3 × 5 min with PBS) and the antibody was applied. Either an antibody directed against the Strep tag (IBA, Göttingen) directly fused to the reporter protein horseradish peroxidase or an antibody directed against the *β*-glucuronidase (rabbit anti-GUSB, Sigma-Aldrich, Munich, Germany) that was detected with a secondary antibody (mouse anti-rabbit) that was connected to horseradish peroxidase was used. After antibody binding, the membrane was washed again (3 × 5 min in PBS) and detection was performed in 20 mL PBS containing 200 *μ*L of 4-chloro-1-naphthol (3% w/v) and 20 *μ*L H_2_O_2_ (30%).

## 6. Results

### 6.1. Inducible Gene Expression for Tagged Protein Purification in *Ms. mazei*

Members of the genus *Methanosarcina* are capable of growth on different substrates including acetate, methanol, methyl amines, and H_2_/CO_2_. This flexibility is reflected in major changes of the transcriptome and proteome upon shift of the growth substrate [14–16]. During the breakdown of methylated amines a series of methyl transferases and corrinoid proteins become active whose genes are downregulated during growth on other substrates [17]. The breakdown of trimethylamine proceeds stepwise where each demethylation step is catalyzed by a different methyl transferase [17–23]. The genes encoding the methyl transferases responsible for the demethylation of trimethylamine and dimethylamine are encoded together in an operon (*mm*1687–*mm*1694) and are highly active in the presence of trimethylamine [17]. In contrast, the breakdown of methanol is independent of the gene products of *mm*1687–*mm*1694 and the transcription of this operon is completely repressed when methanol is used as a growth substrate. Thus, the promoter that controls gene expression of *mm*1687–*mm*1694 seemed a suitable tool to establish a regulatable gene expression system for *Ms. mazei* based on the growth substrate. Jäger et al. [24] have determined the transcriptional start site of *mm*1687 414 bp upstream of the start codon. Therefore, 875 bp of the 5′-untranslated region of the operon *mm*1687–*mm*1694 was chosen for PCR amplification to include the promoter and potential regulatory sequences that could be located upstream of the promoter region. This fragment was termed p1687 and was used for subsequent cloning.

The promoter p1687 was cloned into two potential expression vectors, pWM321 and the pWM321 derivative where the *pac* cassette was replaced by a neomycin resistance cassette (described further below). The vector pWM321 is a shuttle vector that can replicate in both *E. coli* (for cloning purposes) and *Methanosarcina* species [3]. It derives from the naturally occurring *Ms. acetivorans* plasmid pC2A and the *E. coli* cloning vector pBluescript. The R6K ori for pi-dependent replication in *E. coli* derives from the vector pGP704 [7]. However, the pWM321 vector does not contain a promoter system for inducible gene expression in *Methanosarcina* species.

**Figure 1:**
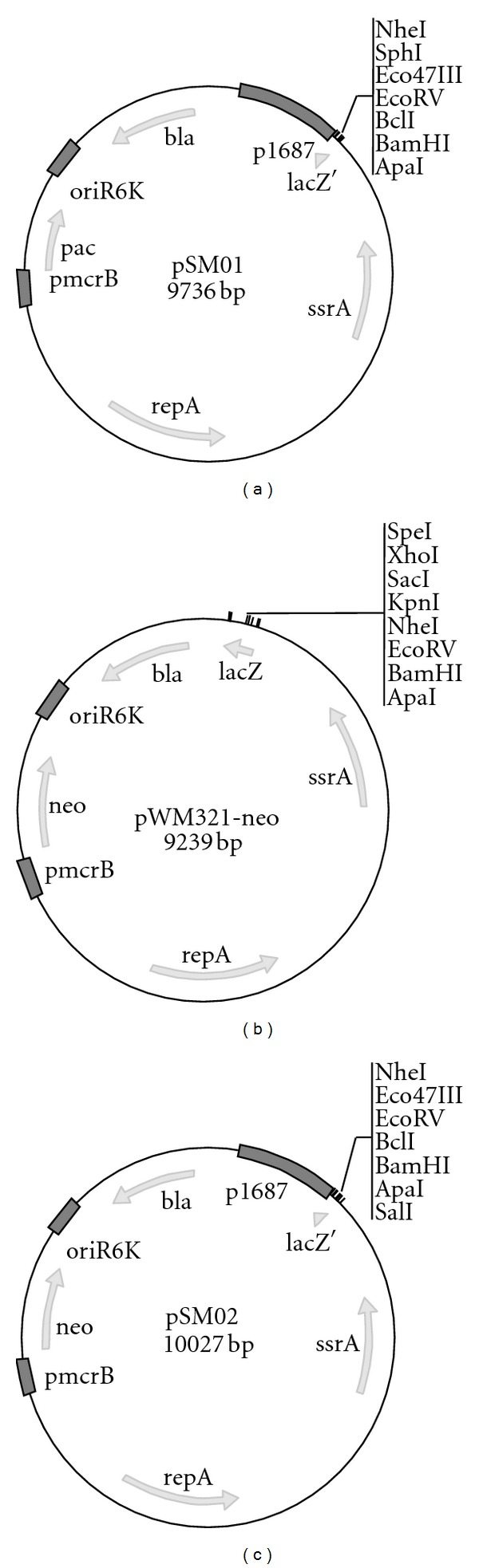
Maps of the plasmids constructed during this study. (a) Plasmid map of pSM01 that is a pWM321 derivative containing in addition the p1687 promoter for inducible protein production. (b) Plasmid map of pWM321-neo that contains the resistance cassette (neo) for neomycin resistance instead of the *pac* gene. (c) pSM02 is a combination of pSM01 and pWM321-neo. It contains both the neomycin resistance cassette and the p1687 promoter for inducible protein production.

**Figure 2:**
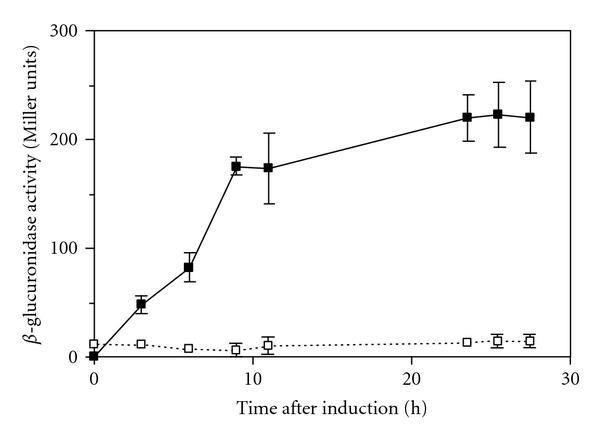
*β*-glucuronidase activity in cell extract of *Ms. mazei* after induction of protein production with trimethylamine. (■) Cultures harboring the plasmid pSM01-uidA-Strep (pWM321-p1687-uidA-Strep). (□) Cultures harboring the plasmid pWM321-uidA without the promoter p1687.

The vector pWM321-neo carrying the p1687 promoter sequence was termed pSM02 (Figure 1(c)), whereas pWM321 containing p1687 was termed pSM01 (Figure 1(a)). Both pSM01 and pSM02 were used for further analyses of inducible gene expression in *Ms. mazei*. The reporter gene *uidA* from *E. coli *together with a Strep-tag encoding sequence at the 3′ end was introduced downstream of the promoter p1687 of pSM01. The resulting plasmid was referred to as pSM01-uidA-Strep and it was found that the activity of *β*-glucuronidase was direct proportional to the transcription of *uidA* that was present under the control of the p1687 promoter. The *Ms. mazei* cultures harboring the pSM01-uidA-Strep plasmid were grown on 30 mM methanol up to an optical density at 600 nm of 0.15. Activity of *β*-glucuronidase was essentially absent under these growth conditions (data not shown). Then *uidA* gene expression was induced by the addition of 50 mM trimethylamine and *β*-glucuronidase activity increased rapidly after induction (Figure 2). After 9 h of induction 175 Miller Units were measured and increased slightly to 220 Miller Units after 30 hours of induction. The control culture contained the pWM321-uidA plasmid that lacked the p1687 promoter. Samples taken from this culture before induction resulted in a *β*-glucuronidas activity of 11.7 ± 3.0 Miller Units and after 30 h induction 10.8 ± 3.9 Miller Units. Hence, the change of growth substrate from methanol to trimethylamine did not affect *β*-glucuronidase production in the culture harboring the promoterless pWM321-uidA plasmid.

**Figure 3:**
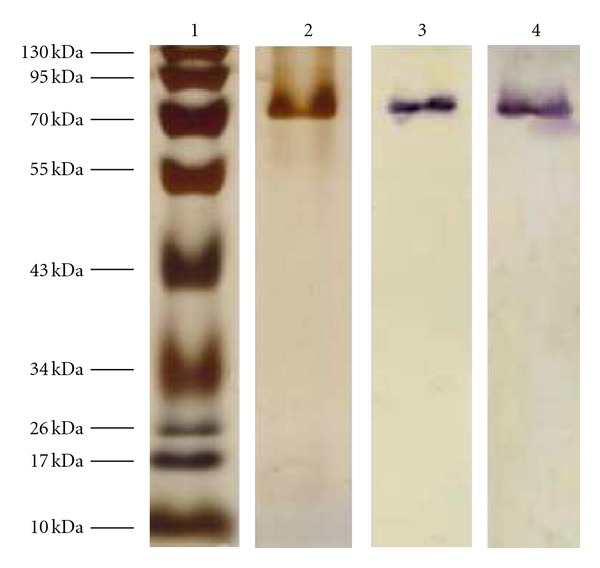
SDS-PAGE of purified *β*-glucuronidase. (1) Fermentas PageRuler molecular mass standard. Elution fraction 5 (0.7 *μ*g) of the *β*-glucuronidase produced in *Ms. mazei* was subjected to SDS-PAGE with subsequent visualization by silver staining (2), Western blot, and antibody-detection with anti-Strep antibody (3) and anti-GUSB antibody (4).

In the next step it was tested whether purification of the reporter protein by affinity chromatography was possible. The use of affinity tags for protein purification is well established in many overproduction systems. From *Ms. mazei*, however, a tagged protein has never been homologously produced and purified so far. The Strep-tag system (IBA, Göttingen, Germany) was chosen as the affinity tag because it allows purification to very high purity and is reasonably resistant against the contents of *Methanosarcina* medium (e.g., reducing agents). The *Ms. mazei* culture harboring the plasmid pSM01-uidA-Strep was grown under the same conditions as the promoter strength investigation and was harvested after 30 h, correlating to the highest *β*-glucuronidase activity to yield the maximum amount of recombinant protein. After lysis of the *Ms. mazei* cells the protein was purified according to the manufacturer instructions. The purity of the *β*-glucuronidase was analyzed by SDS-PAGE and Western blot with antibodies directed against the Strep-tag and *β*-glucuronidase (Figure 3). No difference could be observed between the protein produced in *E. coli* from the plasmid pASK-IBA3-uidA and the protein produced and purified from *Ms. mazei*. Furthermore, the enzyme activity in both preparations was equivalent (59 ± 5 U mg^−1^). The total amount of purified enzyme from *Ms. mazei* was about 0.2 mg per liter of culture.

### 6.2. A Novel Resistance Marker for *Ms. mazei*

**Figure 4:**
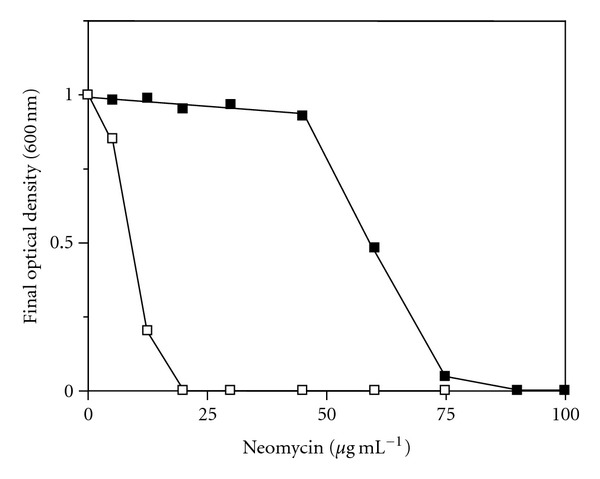
Growth behavior of 5 mL *Ms. mazei* cultures with different concentrations of neomycin. (■) Cultures harboring the pWM321-neo plasmid. (□) Cultures without an additional plasmid.

Working with plasmids in the archaeon *Ms. mazei* is limited by the use of only one antibiotic for selection, puromycin. Most of the antibiotics used for bacteria do not work in archaea because of the differences in cell wall composition and information processing. Furthermore, not all resistance cassettes are suitable for the use in archaeal organisms. We found that the antibiotic neomycin successfully inhibits growth of *Ms. mazei* in a concentration of 20 *μ*g mL^−1^ in the medium (Figure 2). Neomycin is an aminoglycoside that blocks translation and effectively prevents growth of *Ms. mazei* when added to the medium. To investigate whether the inhibitory effect of the antibiotic could be circumvented by the expression of a resistance cassette, a neomycin resistance cassette was introduced into the *E. coli*—*Ms. mazei* shuttle vector pWM321. The puromycin resistance cassette (*pac*) in pWM321 was replaced by the phosphotransferase gene *aph-IIb* from *Pseudomonas aeruginosa* [25] amplified from the vector pBBR-MCS1. This vector is usually selected for kanamycin because the phosphotransferase confers resistance against both kanamycin and neomycin. It catalyzes the ATP-dependent phosphorylation of kanamycin/neomycin [26, 27] and thus inactivates the antibiotic effect [28]. The corresponding vector was named pWM321-neo (Figure 1(b)). Cultures harboring this plasmid were resistant against neomycin at concentrations that prevented growth of the parental strain not harboring the pWM321-neo plasmid (Figure 4). The working concentration of 20 *μ*g mL^−1^ effectively inhibited growth in cultures not harboring the pWM321-neo plasmid whereas cultures containing the pWM321-neo plasmid grew normally up to a neomycin concentration of 50 *μ*g mL^−1^.

Puromycin and neomycin are both translation inhibitors, so it was tested whether the corresponding resistance cassettes would provide overlapping resistance to the other antibiotic. Under the tested conditions (DSM medium 120, growth on trimethylamine or methanol, 5 *μ*g mL^−1^ puromycin, or 20 *μ*g mL^−1^ neomycin) overlapping resistance was not observed so both antibiotics can be used together to select for two different plasmids. These results demonstrate that the neomycin resistance can be used as a novel selection marker in *Ms. mazei* that is compatible with clones already harboring a plasmid or chromosomal deletion with a *pac* resistance cassette.

## 7. Discussion

### 7.1. Inducible Gene Expression in *Ms. mazei*

Inducible expression systems have the advantage that growth phase and protein production phase can be separated. This is often very useful because protein production might slow or stop growth and only minor amounts of cell biomass and recombinant protein may be obtained. In *E. coli* and in many other bacteria inducible protein production systems are well established and routinely used. In archaea, however, many of the systems used in bacteria are not functional due to the differences in metabolism and gene regulation between the two domains. One system that can be used for methanogens of the genus *Methanosarcina* is the pmcr/TetR system of Guss et al. [29]. The authors used the promoter of the gene encoding the key enzyme of methanogeneses, the methyl CoM reductase, and fused it with the bacterial tetracycline operator that allows repressor TetR-mediated induction of gene expression upon the addition of tetracycline. This system was used to inducibly complement deletion mutants but was never used to produce proteins for purification. The same is true for the inducible gene expression system described by Rother et al. [30] that was used for conditional deletion mutagenesis in response to the growth substrate. That study also makes use of a promoter that responds to substrate availability, using the promoter of the methyl transferase involved in methanol degradation (*mtaC1*) of *Methanosarcina acetivorans*. The promoter fusion with the reporter gene *uidA* results in similar *β*-glucuronidase activities during the midexponential growth phase as obtained in this study. However, Rother et al. [30] did not measure induction of gene expression from cells upon switch of growth substrate but measured only *β*-glucuronidase activity of cultures growing on the same substrate for an extended period of time with multiple culture passings. In comparison, our results show that inducible production of a tagged protein is possible during the cultivation of a single culture in 2-3 days and time-consuming culture passings can be avoided.

In accordance with the deep sequencing results of the *Ms. mazei* transcriptome [24] 875 bp of the 5′ untranslated region of the *mm*1687–*mm*1694 operon were chosen as the putative promoter. This sequence is thought to contain the promoter sequence as well as potential binding sites for regulatory proteins. The results of the activity measurements in the cell extracts show that the culture harboring the *uidA* gene fused to this 875 bp sequence showed considerable *β*-glucuronidase activity after induction. *β*-glucuronidase activity was absent during growth on methanol but rapidly increased upon addition of trimethylamine. This means that the 875 bp fragment, termed p1687, indeed directed transcription of the *uidA* gene in response to the availability of trimethylamine. Therefore, the promoter and motifs for regulatory protein binding of the *mm*1687–*mm*1694 operon are present in this 875 bp DNA fragment. Furthermore, the regulation of the promoter is very tight because *β*-glucuronidase activity could not be measured in the cultures before induction with trimethylamine.

The purification of the Strep-tagged *β*-glucuronidase from *Ms. mazei* cell extract led to a pure enzyme preparation that exhibited similar enzyme activities as the enzyme prepared from *E. coli *cell extract. The Strep-tag has proven to be a suitable affinity tag for the anaerobic production of recombinant proteins in *Ms. mazei*.

The yield of recombinant protein purified from the *Ms. mazei* culture was low with 0.2 mg *β*-glucuronidase per liter culture volume and will probably be even lower for the production of proteins containing complex prosthetic groups. However, a much smaller volume of *Ms. mazei* culture will be needed to obtain sufficient amounts of recombinant protein for further analyses compared to classical approaches of protein purification. Furthermore, purification is simple because only a single chromatography step is needed instead of multiple chromatography columns typically employed using the classical approach. This is a great advantage especially for oxygen-sensitive proteins that occur frequently in *Ms. mazei*.

### 7.2. Neomycin Resistance as Novel Selection Marker in *Ms. mazei*

Most antibiotics that act against bacteria are not functional in archaea because of the differences in cellular structure and metabolism. Furthermore, it is estimated that there are only few archaea associated with human life and disease [31] so there is hardly a pharmaceutical or commercial interest to establish antibiotics against archaea. However, certain antibiotics acting against bacteria or used in cell culture of eukaryotic cells might have an effect on archaeal organisms. Furthermore, resistance cassettes exist that may also function or may be optimized for use in archaea. Neomycin is an antibiotic that has been known since 1949 to inhibit bacterial growth [32]. It binds to the ribosomal RNA in the A-site of the bacterial ribosome and promotes improper codon-anticodon binding, leading to misreading of the genetic code and the cessation of the translocation of the ribosome [33, 34]. For some lower eukaryotes, for example, *Saccharomyces cerevisiae* and *Tetrahymena* sp., neomycin has been reported to have the same effect [35, 36]. Outside the domain *Bacteria* neomycin resistance is used as a selective marker in the eukaryote *Tetrahymena thermophila* [37] and the archaeon *Methanococcus maripaludis* [38]. The archaea *Thermoplasma acidophilum* and *Thermococcus celer *are also susceptible to neomycin [39]. However, there is no report about the use of neomycin resistance as a selectable marker in these organisms. It is expected that neomycin does not inhibit growth of halophilic archaea since neomycin does not inhibit growth at typically used concentrations (<500 *μ*M) in high salt concentrations, at least in bacteria [40]. However, the employment of neomycin resistance as a selectable marker in *M. maripaludis* and now *Ms. mazei* suggests that it can be used in other archaea and may expand the genetical toolbox in a variety of organisms. Members of the order Methanosarcinales may be especially susceptible to growth inhibition by neomycin and the plasmid pWM321-neo presented here could be used to confer neomycin resistance to these organisms.
