# Erratum to “A Novel Inducible Protein Production System and Neomycin Resistance as Selection Marker for *Methanosarcina mazei*”

**DOI:** 10.1155/2012/910205

**Published:** 2012-10-21

**Authors:** Sebastian Mondorf, Uwe Deppenmeier, Cornelia Welte

**Affiliations:** Institute for Microbiology and Biotechnology, University of Bonn, Meckenheimer Allee 168, 53115 Bonn, Germany

In the paper “*A novel inducible protein production system and neomycin resistance as selection marker for Methanosarcina mazei*” we stated that there was no inducible protein production system available for methanogenic archaea. However, Macauley et al. [1] have published an acetate-inducible gene expression system for *Methanosarcina acetivorans* that was successfully used to produce a carbonic anhydrase.
